# Concurrent spontaneous coronary dissection and reversible cerebral vasoconstriction syndrome during postnatal care

**DOI:** 10.1186/s43044-024-00464-8

**Published:** 2024-03-18

**Authors:** Sang Gon Yoon, Yeo-Jeong Song, Yun-Seok Song, Jino Park, Seunghwan Kim, Dong-Kie Kim, Ki-Hun Kim, Doo-Il Kim, Hyun Kuk Kim, Dong Ah Lee

**Affiliations:** 1 Division of Cardiology, Department of Internal Medicine, Inje University College of Medicine, Haeundae Paik Hospital, 1435 Jwa-dong, Haeundae-gu, Busan, 48108 Korea; 2 Division of Pulmonology, Department of Internal Medicine, Inje University College of Medicine, Haeundae Paik Hospital, Busan, Korea; 3 Department of Neurology, Inje University College of Medicine, Haeundae Paik Hospital, Busan, Korea

**Keywords:** Spontaneous coronary artery dissection, Reversible cerebral vasoconstriction syndrome, Postpartum, Pregnancy, Case report

## Abstract

**Background:**

Pregnancy-associated spontaneous coronary artery dissection (SCAD) and reversible cerebral vasoconstriction syndrome (RCVS) are rare conditions that may occur during pregnancy and the postpartum period. The coexistence of both diseases may pose a risk to patients, potentially resulting in a variety of complications and clinical manifestations. This is considered the first case of a patient who successfully recovered from a critical condition in the postpartum period, with contemporaneous SCAD and RCVS.

**Case presentation:**

A 33-year-old female with no known medical history was referred to the emergency department after experiencing cardiac arrest, which occurred 1 week after giving birth to her third child. She complained of sudden anterior squeezing chest pain, accompanied by a headache, and eventually collapsed due to ventricular fibrillation with seizure. She was successfully resuscitated after receiving basic life support. She showed an alert mentality and did not experience any further seizure events or additional neurological symptoms. Although vital sign remained stable, the level of highly sensitive troponin I was significantly elevated. Electrocardiography revealed sinus rhythm with T-wave inversion at V1-4, while chest computed tomography (CT) demonstrated severe aspiration pneumonia. The patient was admitted to the intensive care unit due to a high requirement of O2 supply. A consultation with the neurologic department and a brain magnetic resonance angiography (MRA) were conducted for the thunderclap headache. The brain MRA demonstrated stenosis in the basilar artery, the right M2 segment, and bilateral A1 segments, along with sulcal hyperintensity on post-contrast fluid-attenuated inversion recovery (FLAIR) suggesting blood–brain barrier breakdown due to vasoconstriction. Formal echocardiography showed regional wall motion abnormality in the left anterior descending artery (LAD) territory. After the improvement of pneumonia, a coronary angiography was performed, revealing diffuse luminal narrowing from the mid to distal LAD due to a long segmental, extensive dissection. We decided to maintain the medical therapy. A follow-up coronary CT angiography performed 6 months later revealed complete remission of the dissected coronary vessel, and a brain MRA checked 3 months later showed resolved vasoconstriction of the relevant cerebral vessels.

**Conclusions:**

The physicians must be aware of pregnancy-associated complications in certain patients. Clear diagnoses and proper treatments are required in pregnant patients who may be exposed to multiple acute conditions, in order to reduce complications and achieve favorable outcomes.

## Background

Pregnancy-associated spontaneous coronary artery dissection (SCAD) and reversible cerebral vasoconstriction syndrome (RCVS) are rare complications that may occur during pregnancy and the postpartum period [1, 2]. The causes of pregnancy-associated SCAD and RCVS are still unknown. The presumed causes of SCAD and RCVS, which can result in the impairment of arterial tone, are the hemodynamic stress due to an increase in cardiac output and hormonal changes related to the degeneration of the vessel walls [1, 3, 4]. In addition, there is supporting evidence of vascular muscle cell and endothelial dysfunction in relevant vessels in both diseases [5–7]. The concurrent presence of both diseases may pose a risk to patients, resulting in numerous complications and clinical manifestations. This is considered the first case of a patient who successfully recovered from a critical condition in the postpartum period, with contemporaneous SCAD and RCVS.

## Case presentation

**Fig. 1:**
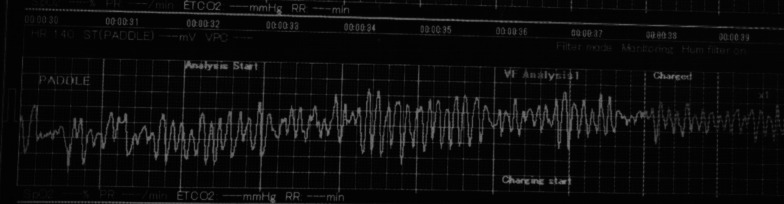
Ventricular fibrillation was documented in real-time monitoring during the transfer to the hospital

**Fig. 2:**
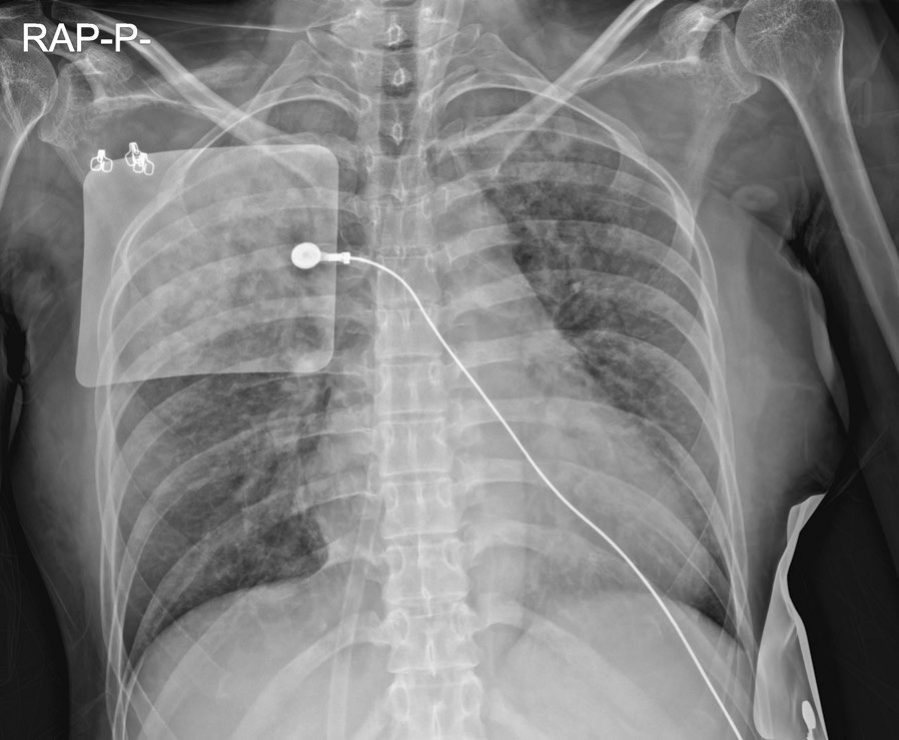
Initial chest X-ray reveals diffuse haziness with infiltration throughout the entire lung fields and prominent cardiomegaly

**Fig. 3:**
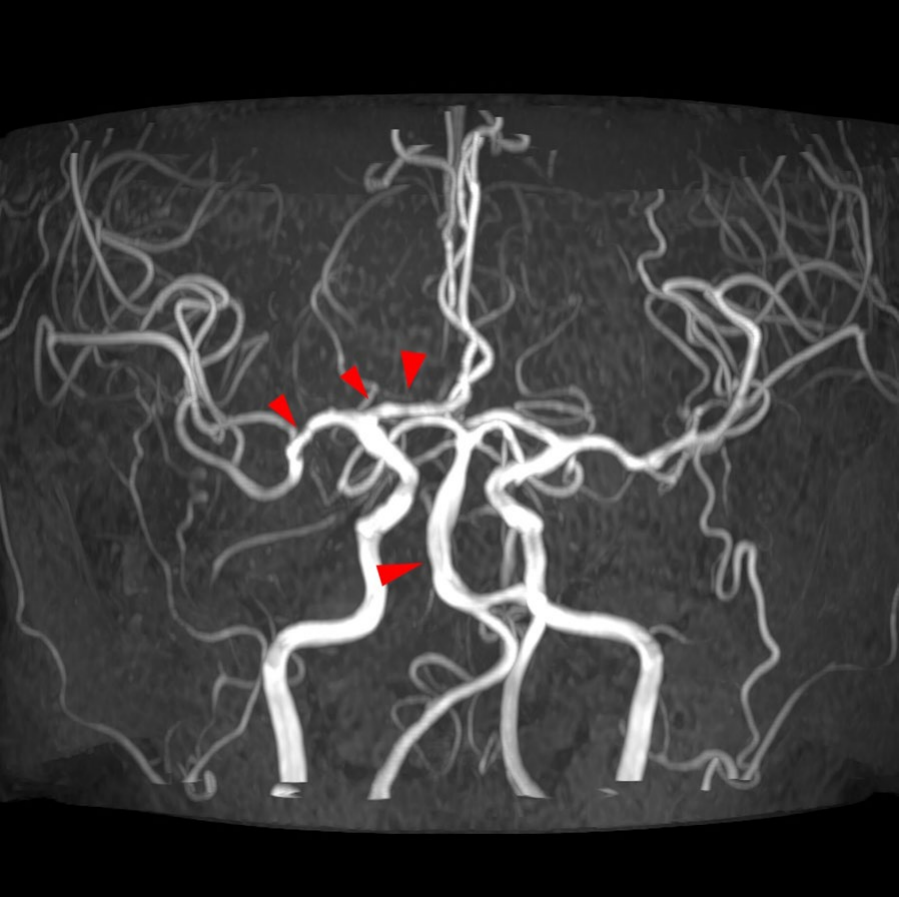
Brain MRA showed luminal stenosis in the basilar artery, the right M2 segment, and bilateral A1 segments

**Fig. 4:**
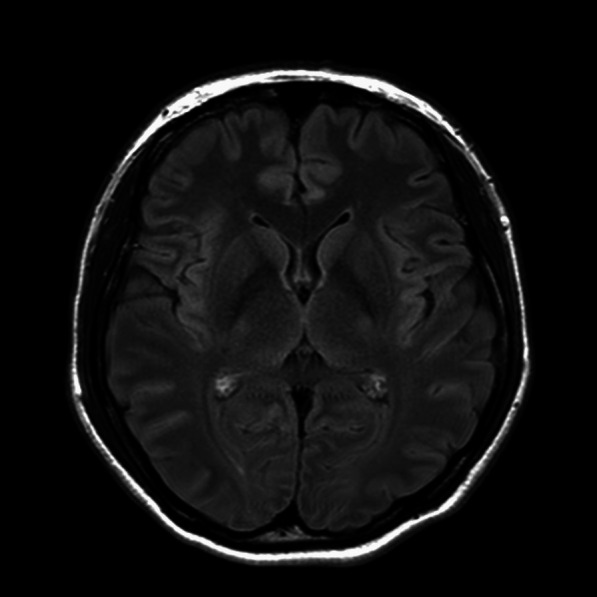
Sulcal hyperintensity on post-contrast FLAIR implying blood–brain barrier breakdown due to vasoconstriction and contrast exposure

**Fig. 5:**
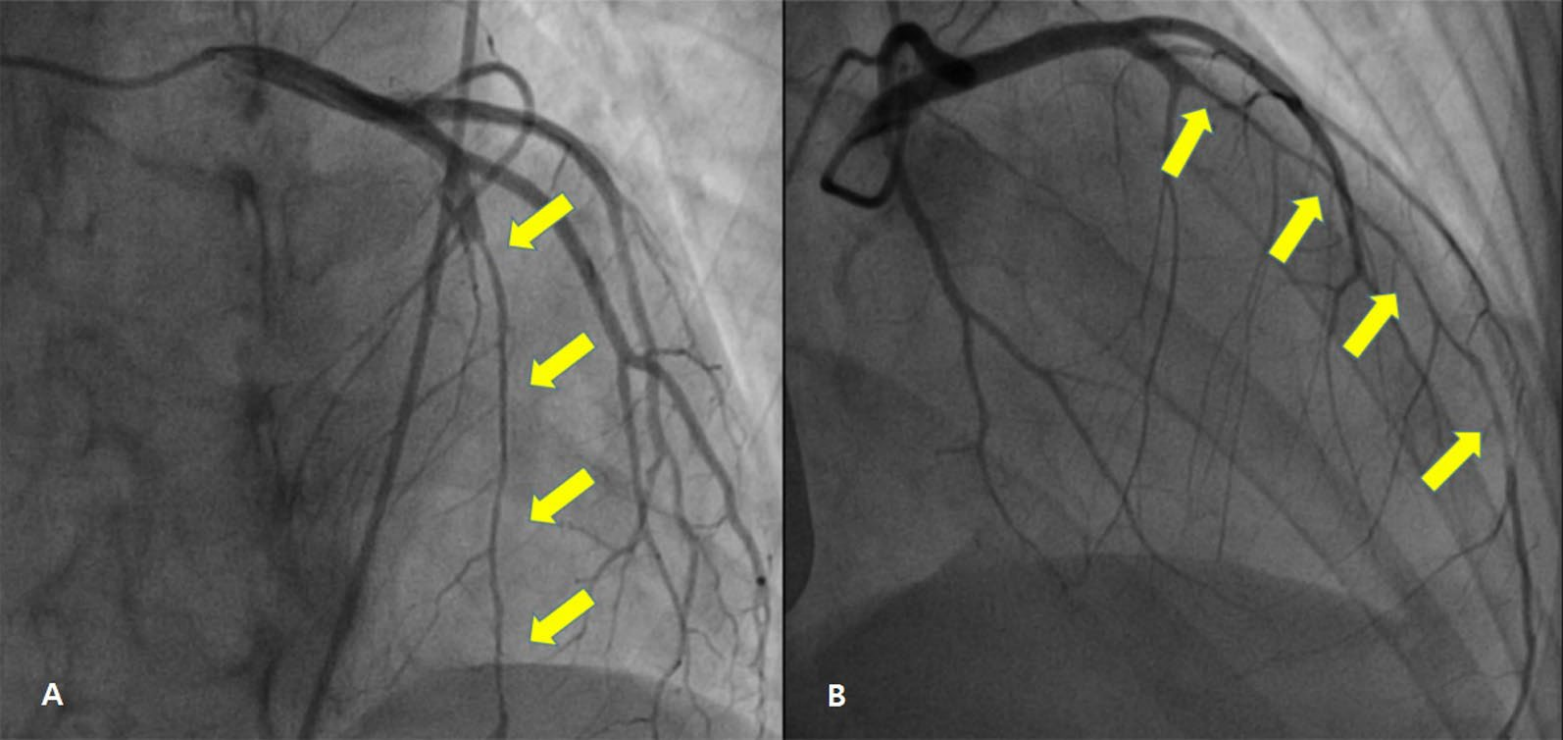
CAG [LAO cranial view (**A**) and RAO cranial view (**B**)] demonstrating diffuse luminal narrowing from mid to distal LAD due to long segmental huge dissection with thrombolysis in myocardial infarction (TIMI) III flow

**Fig. 6:**
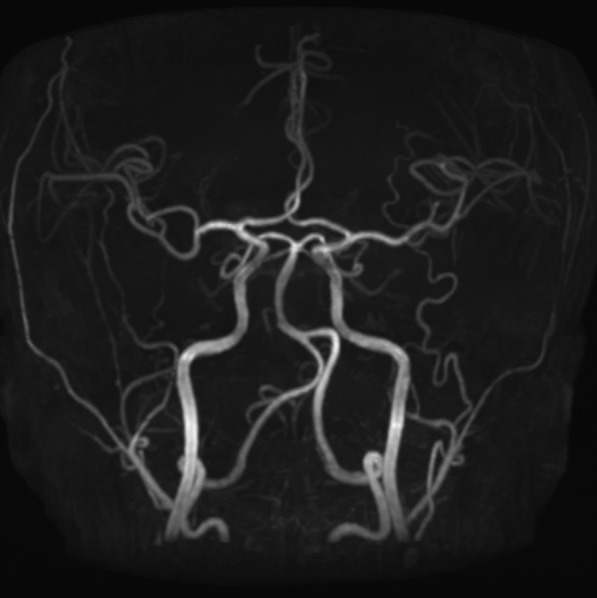
Follow-up brain MRA showing resolution of vasoconstriction without any complications of posterior reversible encephalopathy syndrome (PRES), seizure, hemorrhage, and brain infarction

**Fig. 7:**
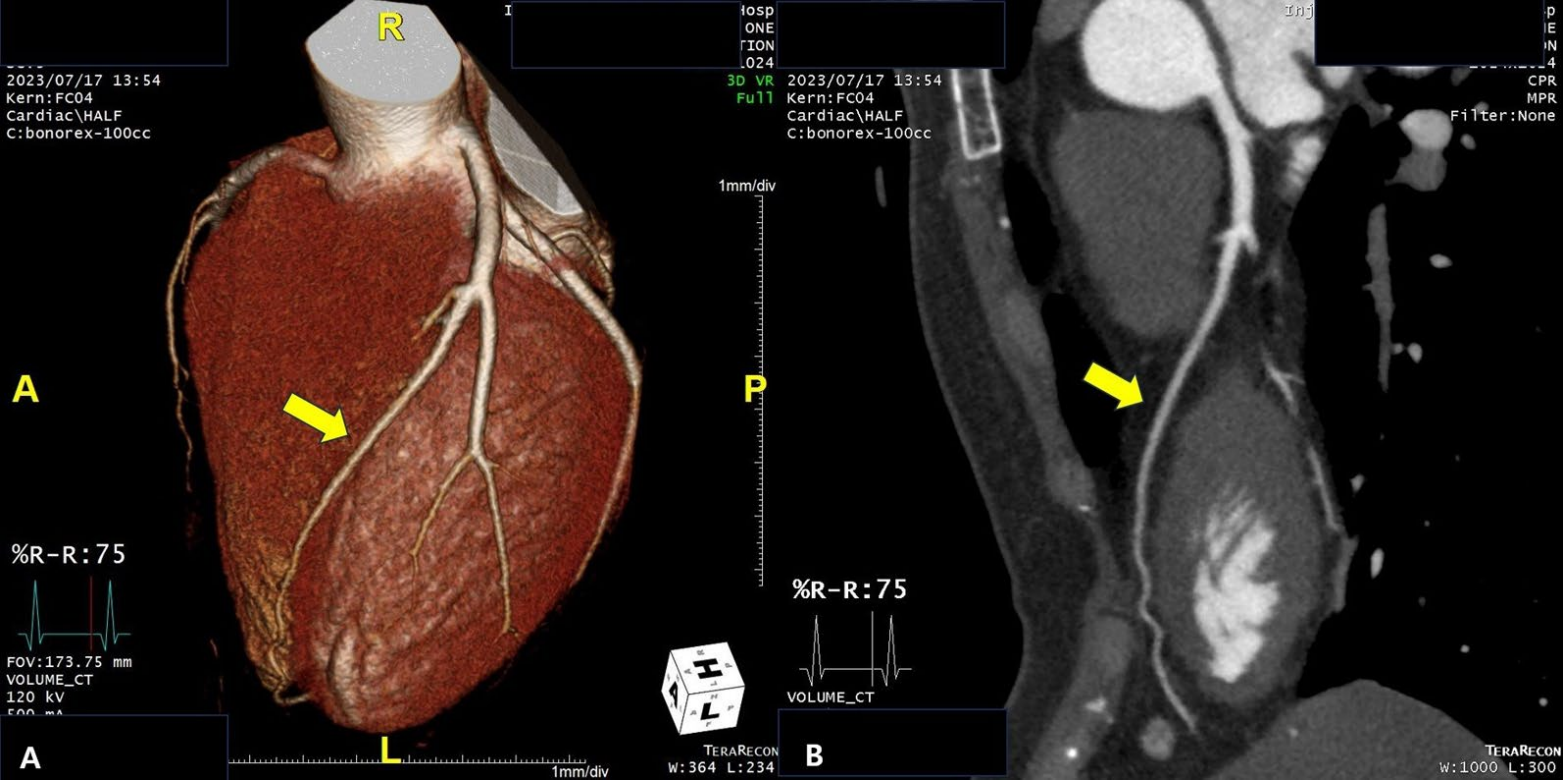
Follow-up coronary CT angiography [3D rendered view (**A**), 2D view (**B**)] of well healed LAD showing near normal coronary artery finding

A 33-year-old-female with no underlying diseases was referred to the emergency department due to cardiac arrest. She had been recovering at a postpartum care center since giving birth to her third child a week ago. On the day she visited the hospital, she complained of sudden squeezing chest pain, headache, and collapsed with a seizure. The chest pain was the first attack; however, the recurring severe headache had started three days before the initial visit. Ventricular fibrillation, as shown in Fig. 1, was documented in real time monitoring performed by rescue workers. Basic life support was immediately conducted with two electrical shocks, resulting in successful resuscitation. On the arrival to the hospital, she showed an alert mentality and did not experience any further seizure events or other neurological symptoms. Additionally, the initial brain compute tomography (CT) without enhancement revealed no abnormal findings. Blood pressure was 113/83 mmHg, heart rate was 114 beats/min, respiratory rate was 26 breaths/min, and blood temperature was 36.6 °C on the initial visit. The highly sensitive troponin I level was slightly increased to 137.2 pg/ml, and creatinine kinase myocardial band was 2.1 ng/ml, which jump up to a maximum of 1588.7 pg/ml and 9.9 pg/ml, respectively. The initial pro-brain natriuretic peptide level was 306.2 pg/mL. The electrocardiography (ECG) showed sinus rhythm with T-wave inversion at V1–V4. Prominent cardiomegaly and severe haziness were observed in both lungs (Fig. 2). Therefore, we conducted a chest computed tomography, which revealed a diffuse consolidation ground-glass opacity pattern consistent with the findings of aspiration pneumonia combined with pulmonary edema. Due to high risk of recurrent cardiac arrest and increase requirement for oxygen supply, she was admitted to the intensive care unit. A neurologic consultation was conducted, and brain magnetic resonance angiography (MRA) was performed due to intermittent thunderclap headache, which strongly suggested the features of RCVS. Brain MRA showed stenosis in the basilar artery, the right M2 segment, and bilateral A1 segments (Fig. 3). Additionally, sulcal hyperintensity was observed on post-contrast fluid-attenuated inversion recovery (FLAIR), indicating blood–brain barrier breakdown which resulted from vasoconstriction and contrast exposure (Fig. 4). Formal echocardiography demonstrated a regional wall motion abnormality of the left anterior descending artery (LAD), grade I diastolic dysfunction, as well as mild left ventricular systolic dysfunction characterized by an ejection fraction of 48%. The patient did not have any ongoing cardiac symptoms, but only experienced recurrent headaches. Her follow-up laboratory test showed the stabilization of cardiac markers. Coronary angiography (CAG) was performed via the right femoral artery six days after admission, when the pneumonia had remarkably improved, due to severe radial artery spasm. Intracoronary nitroglycerin 200mcg was applied twice, following the anteroposterior caudal view which showed a significant diffuse long lesion in the LAD. Post-injection angiography (Fig. 5) revealed persistent diffuse luminal narrowing from the mid to distal LAD, which was attributed to a long segmental huge dissection with Thrombolysis in Myocardial Infarction (TIMI) 3 flow. This condition was assessed as SCAD. Since the patient was stable, we decided to continue administrating medical treatment. The calcium channel blockers (CCBs) including nimodipine with low-dose diltiazem, low-dose nebivolol, candesartan, aspirin, and a combination of ezetimibe and atorvastatin were given. A follow-up brain MRA (Fig. 6) checked 3 months later, showed complete resolution of vasoconstriction in the relevant cerebral vessels. Additionally, a coronary CT angiography performed 6 months later which confirmed the full recovery of the coronary vessel (Fig. 7). So far, the patient is free from the related symptoms.

## Discussion

The SCAD is a rare cause of acute coronary syndrome or sudden cardiac death, specifically associated with pregnancy or the postpartum period in young women [8, 9]. It is defined as a non-atherosclerotic and non-traumatic separation of the arterial wall layers, resulting in the development of a false lumen which can cause significant collapse of the true lumen [1]. The short-term mortality rate is relatively high, reaching approximately 38%. However, the long-term outcome is favorable after surviving from the initial event [9]. Pregnancy-associated SCAD is associated with more severe symptoms than general SCAD. It is known to occur frequently during the first month of the postpartum period and is more commonly observed among multiparous women, particularly those over the age of 30 [10]. It often involves multivessel coronary arteries involving LAD [8]. The main diagnostic method in patients suspicious of acute coronary syndrome due to coronary dissection is coronary angiography, which should be performed as soon as possible [11]. There are currently no optimal treatment guidelines for pregnancy-associated SCAD. Conservative therapy may be considered for benign cases. Percutaneous coronary intervention should be applied to patients with ongoing ischemia, those with hemodynamic instability, or those with focalized single vessel with significant flow limitations. In cases of left main or multivessel proximal LAD involvement with hemodynamic instability, the consideration of coronary artery bypass grafting surgery should be taken into account [1, 11]. The overall prognosis is determined in accordance with the amount of viable myocardium, the extension of the dissection, and the overall clinical status [8].

The RCVS is an uncommon clinical condition, with a higher prevalence observed in young women during the puerperium period, reported to be around 7–9%. This condition is characterized by recurrent thunderclap headaches with or without other neurological symptoms, along with reversible multifocal cerebral vasoconstriction of medium-sized cerebral vessels [2]. The acute headache may be accompanied by vomiting, nausea, photophobia, phonophobia, and in some cases, seizures [12]. The diagnosis of RCVS is based on consistent clinical characteristics, physical examination, and neurovascular imaging with CT or MRA, revealing multifocal cerebral vasoconstrictions [2, 4, 13]. Furthermore, it is recommended to undergo a follow-up imaging test to demonstrate complete or at least significant improvement of vasoconstriction [12, 14]. Approximately 25–33% of patients may experience complications such as seizures, ischemic stroke, brain hemorrhage, and posterior reversible encephalopathy syndrome according to previous reports [2, 12]. The majority of patients with RCVS show a self-limiting course, resulting in full recovery or only a few sequelae. The symptom of a headache usually improves within 3 weeks, while vasoconstriction in imaging tests typically improves within 3 months [2, 12, 15]. The patients require close monitoring for potential complications and, if experiencing symptoms, they should receive prompt medical treatment, which may include analgesia, antiepileptics, and antiemetics if necessary. Nimodipine, among the CCBs, is the preferred medication for relieving headaches. Additionally, verapamil and nicardipine may be considered as alternative medications [16, 17].

The etiologies of concurrent pregnancy-associated SCAD and RCVS remain unclear in this case. One possible explanation may be physiological adaptation following pregnancy. There are reports suggesting that vascular shear stress increases in the end of pregnancy due to a 30–50% increase in plasma volume, which consequently leads to an increase in cardiac output. During labor, active Valsalva efforts may partly contribute to sympathetic overactivity and stress in coronary and cerebral vessels, potentially precipitating SCAD with RCVS [1, 2, 10, 14]. Furthermore, pregnancy induces changes in the levels of progesterone and estrogen, which are elevated during the term and rapidly decrease during the postpartum period. The hormonal changes may affect the vascular endothelium, which has hormone receptors [18]. In addition, exposure to high levels of hormones during pregnancy may result in the degeneration of vessel walls, leading to impairments in arterial tone, vascular cells, and endothelial function [1–3]. However, the exact mechanisms have yet to be clearly identified.

Several cases of extracerebral vascular involvement in RCVS have been previously described, including associations with carotid artery dissection or renal artery spasms. While there have been only a few reports of coronary vasospasm with RCVS, it is well known that cardiac involvement is extremely rare in RCVS [19–21]. On the other hand, SCAD with extracoronary involvement is well correlated with vascular abnormalities such as aneurysm, pseudoaneurysm, fibromuscular dysplasia, and dissection. However, there is no mention of RCVS [22, 23]. The simultaneous existence of both diseases may pose a risk to patients and lead to various complications. Therefore, receiving a quick diagnosis followed by appropriate treatment will lead to a positive outcome.

## Conclusions

This case demonstrates the concurrent occurrence of pregnancy-associated SCAD and RCVS in a single patient, which is an exceptionally rare condition. Although pregnancy is an important natural process, the physicians need to remain alert as the physiologic changes associated with pregnancy can lead to various complications in certain patients, requiring careful attention. Therefore, thorough decision-making, accurate diagnoses, and proper treatments are required in pregnant patients who may have multiple concurrent acute diseases in order to reduce the overall risk of complications and achieve favorable outcomes.

## Data Availability

All data generated or analyzed (and its supplementary files) during the admission of this patient are available in this manuscript.

## Abbreviations

CAG: Coronary angiography
CCB: Calcium channel blocker
CT: Computed tomography
ECG: Electrocardiography
FLAIR: Fluid-attenuated inversion recovery
LAD: Left anterior descending artery
MRA: Magnetic resonance angiography
RCVS: Reversible cerebral vasoconstriction syndrome
SCAD: Spontaneous coronary artery dissection
TIMI: Thrombolysis in myocardial infarction
